# Characterization of *Angraecum* (Angraecinae, Orchidaceae) Plastomes and Utility of Sequence Variability Hotspots

**DOI:** 10.3390/ijms25010184

**Published:** 2023-12-22

**Authors:** Cheng-Yuan Zhou, Wen-Jun Lin, Ruyi Li, Yuhan Wu, Zhong-Jian Liu, Ming-He Li

**Affiliations:** 1 Key Laboratory of National Forestry and Grassland Administration for Orchid Conservation and Utilization at Landscape Architecture and Arts, Fujian Agriculture and Forestry University, Fuzhou 350002, China; zcy810338055@126.com (C.-Y.Z.); ichhabeihn@163.com (R.L.); 15280838312@163.com (Y.W.); 2 Fujian Colleges and Universities Engineering Research Institute of Conservation and Utilization of Natural Bioresources, Fujian Agriculture and Forestry University, Fuzhou 350002, China; linwenjun123@126.com

**Keywords:** Darwin’s orchid, *Angraecinae*, Orchidaceae, plastid genome, phylogenetic analysis

## Abstract

*Angraecum*, commonly known as Darwin’s orchid, is the largest genus of Angraecinae (Orchidaceae). This genus exhibits a high morphological diversity, making it as a good candidate for macroevolutionary studies. In this study, four complete plastomes of *Angraecum* were firstly reported and the potential variability hotspots were explored. The plastomes possessed the typical quadripartite structure and ranged from 150,743 to 151,818 base pair (bp), with a guanine–cytosine (GC) content of 36.6–36.9%. The plastomes all contained 120 genes, consisting of 74 protein-coding genes (CDS), 38 transfer RNA (tRNA) genes and 8 ribosomal RNA (rRNA) genes; all *ndh* genes were pseudogenized or lost. A total of 30 to 46 long repeats and 55 to 63 SSRs were identified. Relative synonymous codon usage (RSCU) analysis indicated a high degree of conservation in codon usage bias. The Ka/Ks ratios of most genes were lower than 1, indicating that they have undergone purifying selection. Based on the ranking of Pi (nucleotide diversity) values, five regions (*trnS^GCU^*-*trnG^GCC^*, *ycf1*-*trnN^GGU^*, *trnN^GUU^*-*rpl32*, *psaC*-*ndhE* and *trnS^GCU^*-*trnG^GCC^*) and five protein-coding genes (*rpl32*, *rps16*, *psbK*, *rps8*, and *ycf1*) were identified. The consistent and robust phylogenetic relationships of *Angraecum* were established based on a total of 40 plastomes from the Epidendroideae subfamily. The genus *Angraecum* was strongly supported as a monophyletic group and sister to Aeridinae. Our study provides an ideal system for investigating molecular identification, plastome evolution and DNA barcoding for *Angraecum*.

## 1. Introduction

Darwin’s most famous hypothesis was that a hawkmoth with an 11-inch (approximately 28 cm) proboscis would pollinate the Madagascan orchid [1]. This orchid belongs to *Angraecum*, commonly known as Darwin’s orchid, and is the largest genus of Angraecinae (Orchidaceae), comprising over 220 species [2,3]. Members of the *Angraecum* genus are mainly distributed from Madagascar to Africa and the Mascarene Islands [4]. Madagascar is the diversity center of this genus with approximately 142 species, 90% of which are endemic [5]. This genus is primarily characterized by its white to green flowers, labellum with a clavate or filiform spur, and two pollinia [6]. The high morphological variation among *Angraecum* species makes it one of the most valuable ornamental orchids. Approximately 190 artificial interspecific hybrids of *Angraecum* have been produced and registered with the Royal Horticultural Society (http://apps.rhs.org.uk/horticulturaldatabase/orchidregister/orchidregister.asp, accessed on 2 November 2023). Additionally, this genus also plays a critical role in pollination biology, evolution and ecological research [7,8,9].

Due to its extensive morphological diversity, this genus has generally been considered one of the most complicated groups within Angraecinae since its establishment. Morphologically, Garay proposed 19 sections to categorize the *Angraecum* species based on their floral characters [10]. However, recent molecular phylogenetic studies have revealed that *Angraecum* was non-monophyletic, and most of the sections defined by Garay were also non-monophyletic [8,11,12,13,14]. Additionally, all studies consistently showed unstable topologies with weak to moderate support. It seems that a limited number of traditional molecular markers have hindered our understanding of *Angraecum* phylogeny, making it challenging to distinguish between different *Angraecum* species. Exploring the molecular phylogenetic markers with more loci suitable for *Angraecum* is needed.

Advancements in next-generation sequencing (NGS) technology have greatly facilitated the acquisition of complete plastomes, which can provide more loci to clarify the phylogenetic relationships of complex taxa [15,16,17]. Plastomes are suitable for phylogenetic analysis due to their uniparental inheritance, abundance of informative loci and moderate mutation rate [18]. In recent years, whole plastome data have significantly advanced our understanding of the relationships within Orchidaceae [19,20,21]. Liu et al. used plastome sequences to elucidate the phylogenetic relationships within the *Cleisostoma*–*Gastrochilus* clades, revealing strong support and a stable topological structure [22]. Based on 79 protein-coding sequences of 46 species from 16 genera, Tu et al. showed a robust phylogenetic framework of the *Cheirostylis* and *Goodyera* clades of Goodyerinae [23]. Moreover, the comparison of plastome structures has proven valuable in understanding the molecular evolutionary patterns involving gene duplication, loss, rearrangement, and transfer within Orchidaceae [24,25,26,27,28]. However, no studies of *Angraecum* plastomes have been reported, hindering our understanding of the plastome evolution and phylogenetic relationships of this genus.

To enhance our understanding of *Angraecum* plastome characteristics, structural diversity and evolution, we firstly present four *Angraecum* plastomes. This study aims to evaluate variations in high-variability sites and simple sequence repeats, characterizing and contrasting *Angraecum* plastomes in order to understand the evolutionary pattern of the plastome and resolving phylogenetic relationships in *Angraecum* for accurate authentication of *Angraecum* species.

## 2. Results

### 2.1. Characteristics of the Plastome

**Figure 1:**
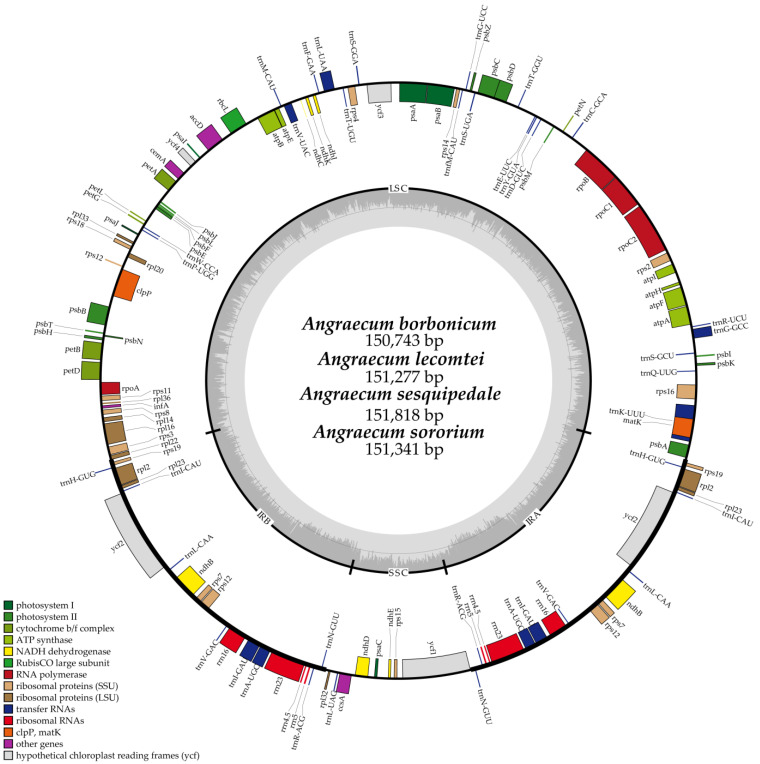
The annotation map of *Angraecum* plastome. The darker gray in the inner circle corresponds to GC content. The IRA and IRB (two inverted repeating regions), LSC (large single-copy region), and SSC (small single-copy region) are indicated outside the GC content.

A total of four newly sequenced *Angraecum* plastomes comprised an LSC region (87,889–88,904 bp), an SSC region (11,599–11,922 bp) and a pair of IRs (25,387–25,982 bp) (Figure 1). Plastome sizes ranged from 150,743 bp (*A. borbonicum*) to 151,818 bp (*A. sesquipedale*). Each *Angraecum* plastome possessed the quadripartite structure with similar percentages in each region (LSC 58.1–58.6%, IR 16.8–17.1%, and SSC 7.7–7.9%) (Table 1). The GC content of the whole plastome exhibited minimal variation, ranging from 36.6% to 36.9%.

**Figure 2:**
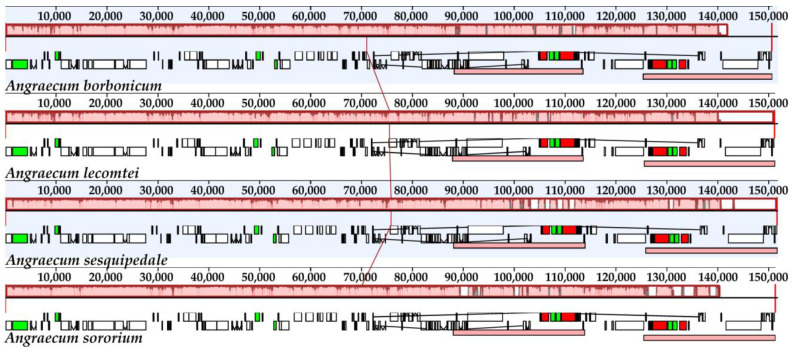
Plastome comparison of four species of *Angraecum* using a progressive MAUVE algorithm. The locally collinear blocks are represented by blocks of the same color connected by lines. Genome regions are color-coded as CDS, tRNA, rRNA, and non-coding region.

All four plastomes of *Angraecum* encoded 120 genes, encompassing 74 CDSs, 38 tRNA genes and 8 rRNA genes (Table 1). Among them, 18 genes were replicated in the IR regions, comprising 5 protein-coding genes (*rpl2*, *rpl23*, *rps7*, *rps19* and *ycf2*), 4 rRNA genes (*rrn4.5*, *rrn5*, *rrn16*, and *rrn23*), and 8 tRNA genes (*trnA^UGC^*, *trnH^GUG^*, *trnI^CAU^*, *trnI^GAU^*, *trnL^CAA^*, *trnN^GUU^*, *trnR^ACG^*, and *trnV^GAC^*) (Figure 1). The loss or pseudogenization of the *ndh* genes was widespread among all *Angraecum* plastomes (Figure 1, Table 1). All four plastomes shared the same pseudogenes (*ndhB*/*C*/*D*/*E*/*J*/*K*). No significant rearrangements among these plastomes were detected by a Mauve analysis (Figure 2).

**Figure 3:**
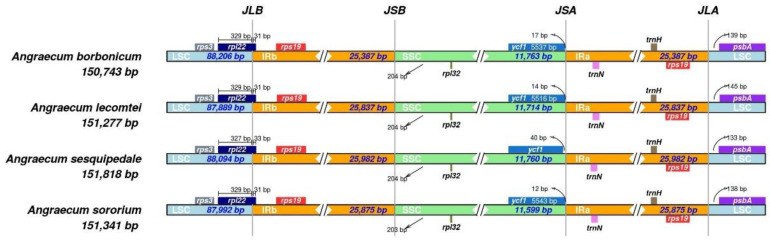
Comparison of junctions between the LSC (large single-copy region), SSC (small single-copy region), and IR (inverted repeat regions) regions among four *Angraecum* plastomes.

The comparative analysis of plastome boundary genes for four *Angraecum* plastomes revealed a highly conserved distribution pattern (Figure 3). The *rpl22* gene in all species spanned from LSC to IRb, with a length of 31 bp to 33 bp. The *ycf1* gene was entirely located in the SSC region, and there were no *ycf1* fragments near the junction between the IRb and the SSC (JSB). For the junction between the IRa and the LSC (JLA), the *trnH* and *psbA* genes were detected.

### 2.2. Repeated Analysis

**Figure 4:**
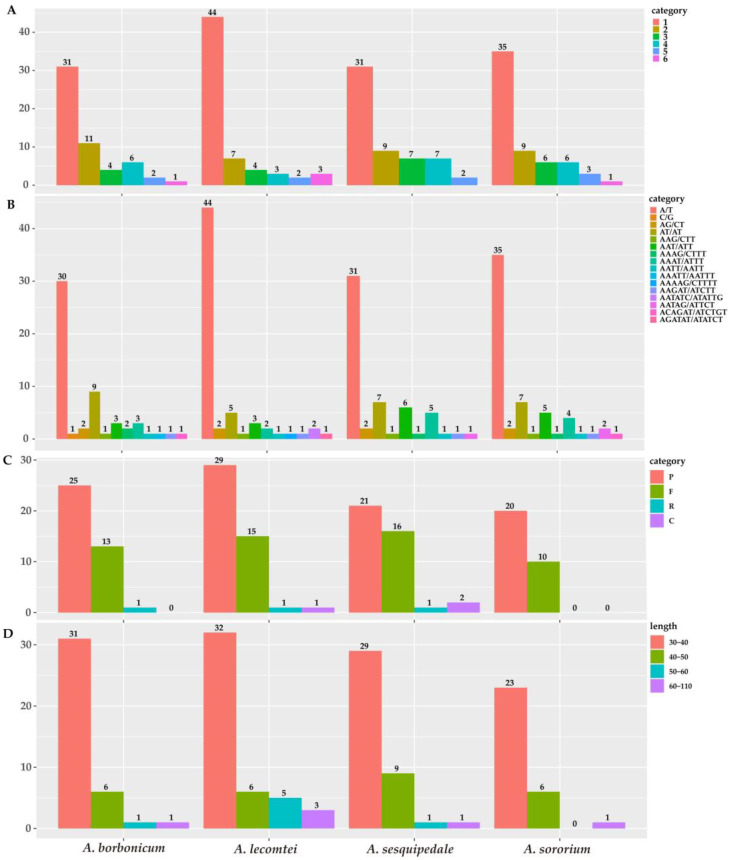
Summary of sequence repeats across the *Angraecum* plastomes. (**A**) Frequency of identified SSR motifs (mono-, di-, tri-, tetra-, penta- and hexa-); (**B**) Frequency of classified repeat types (considering sequence complementary); (**C**) Variation in repeat abundance and type (P, palindromic; F, forward; R, reverse; C, complement); (**D**) The number of long repeats sequences by length.

Six types of simple sequence repeats (SSRs) (mononucleotide, dinucleotide, trinucleotide, tetranucleotide, pentanucleotide, and hexanucleotide) were examined to explore potential genetic markers suitable for clarifying intragenus variations in *Angraecum*. All categories were detected, with a total of 55 (*A. borbonicum*) to 63 (*A. lecomtei*) SSRs (Figure 4, Supplementary Table S2). Mononucleotide repeats were the most frequent type, followed by dinucleotide repeats, with a range of 7 (*A. lecomtei*) to 11 (*A. borbonicum*). Among these classified repeat types, the A/T mononucleotide repeats were the most frequently observed, with a range of 30 (*A. borbonicum*) to 44 (*A. lecomtei*).

A total of 155 long repeats were detected in *Angraecum* plastomes, comprising 4 types of long repeats (palindrome, forward, reverse, and complement) (Figure 4, Supplementary Table S3). Among them, all types were detected within two species (*A. lecomtei* and *A. sesquipedale*), three types were detected within *A. borbonicum*, and only two types were detected within *A. sororium* (Figure 4). The number of long repeats ranges from 30 (*A. sororium*) to 46 (*A. lecomtei*) (Figure 4, Supplementary Table S3). Palindrome repeats were the dominant type of long repeats, followed by forward. The length of long repeats in all species mostly ranged from 30 bp to 40 bp. There were two extremely long repeat sequences found within *A. lecomtei*, 104 bp and 80 bp, respectively.

### 2.3. Codon Usage Analyses

**Figure 5:**
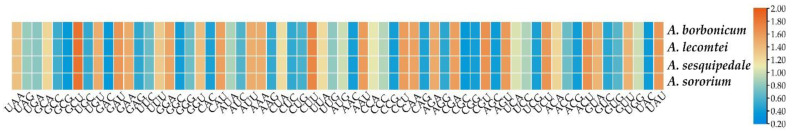
The RSCU (relative synonymous codon usage) values of concatenated 68 CDSs for four plastomes. Color key: the red values mean higher RSCU values and the blue values mean lower RSCU values.

The concatenated sequences of 68 CDSs (*ndh* genes were widespread pseudogenized or lost) were used to calculate the RSCU values and codon usage frequency of *Angraecum* plastomes. Visualization of the RSCU values for *Angraecum* plastomes revealed a highly conserved codon usage bias (Figure 5, Supplementary Table S4). All species possessed 64 different types of codons and encoded a total of 19,377–19,389 codons. Among these codons, leucine (Leu) was the most frequent amino acid, while cysteine (Cys) had the lowest frequency (Supplementary Table S4). The codon GCU exhibited the highest RSCU value, while the codon CGC had the lowest RSCU value. The most frequently used stop codon was UAA, and then UAG and UGA.

### 2.4. Selective Pressure Analysis

**Figure 6:**
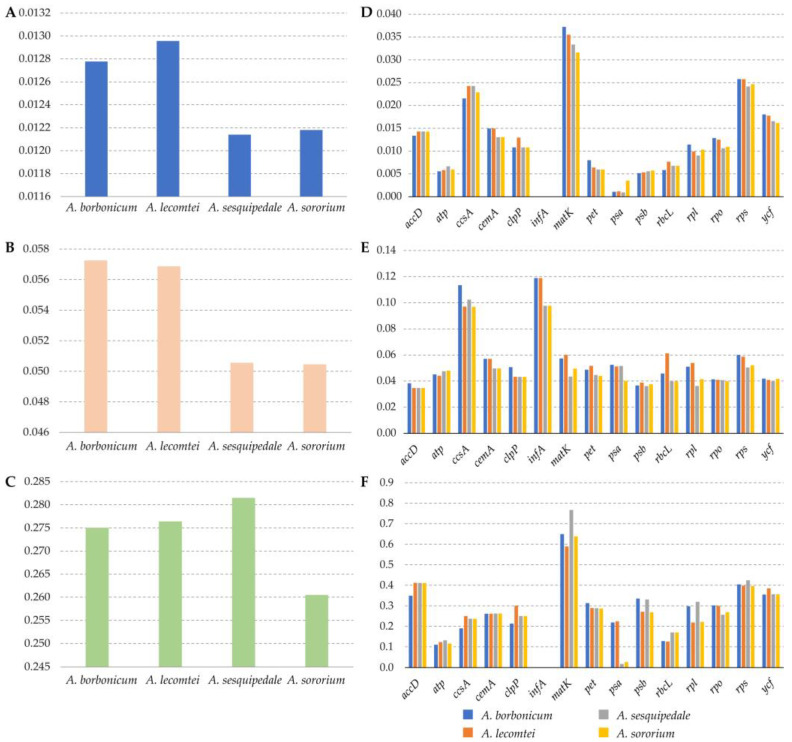
Selective pressure of shared protein-coding genes in four *Angraecum* species. (**A**–**C**) Ka, Ks, and Ka/Ks values of four *Angraecum* plastomes. (**D**–**F**) Ka, Ks, and Ka/Ks of different genes or gene groups in four *Angraecum* plastomes.

The nonsynonymous (Ka), synonymous substitution rates (Ks), and the ratio Ka/Ks were calculated to explore whether the protein-coding genes of four *Angraecum* plastomes have undergone selection (Figure 6, Supplementary Table S5). *A. borbonicum* and *A. lecomtei* exhibited relatively high Ka and Ks values among the four *Angraecum* plastomes. *A. sesquipedale* had the highest average Ka/Ks value (0.2815). The genes *atpH*, *infA*, *psaC*, *psaJ*, *psbC*, *psbE* and *psbI* were found to undergo neutral evolution (Ka/Ks = 1). The majority of the protein-coding genes were found to have undergone purifying selection. (Ka/Ks < 1).

### 2.5. Plastome Sequence Divergence and Barcoding Investigation

**Figure 7:**
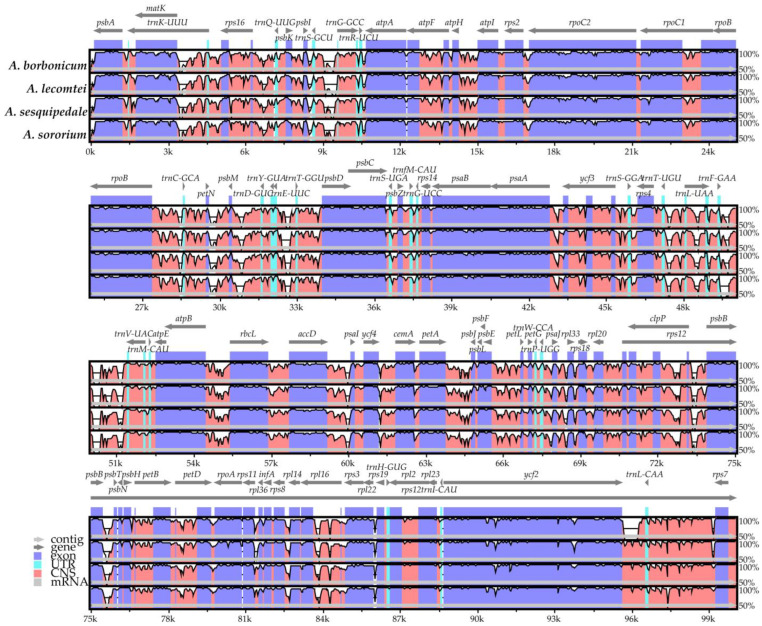
Global alignment of four *Angraecum* plastomes by mVISTA with *Thrixspermum centipeda* as a reference. The *y*-axis shows the coordinates between the plastomes.

The plastome divergence among *Angraecum* species was calculated using an mVISTA program with the annotated plastome of *Thrixspermum centipeda* as a reference genome (Figure 7). The results showed that the greatest variation was localized within the LSC and SSC regions, whereas the IR regions exhibited higher conservation. The coding regions were highly conserved in comparison to the non-coding regions. These results indicate that several regions may be suitable for DNA barcodes that can distinguish different *Angraecum* species easily.

**Figure 8:**
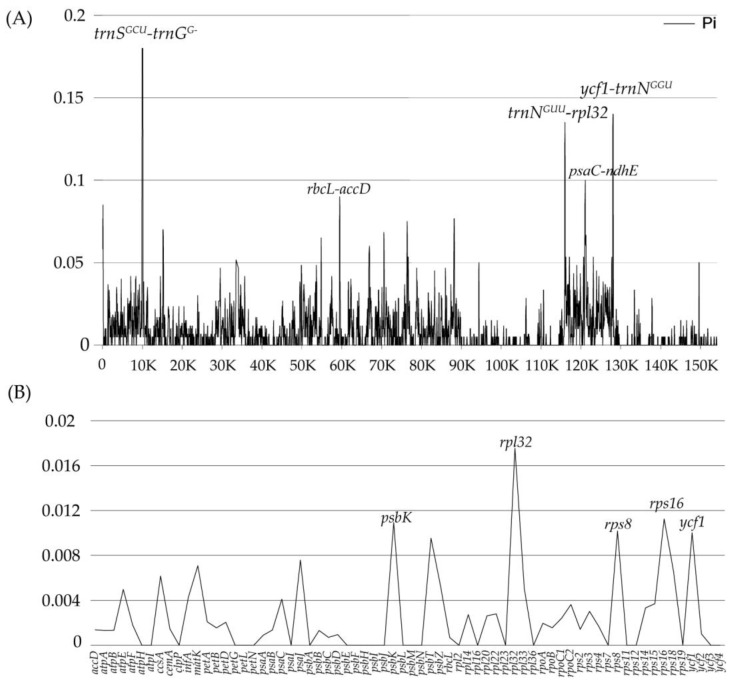
Sliding window test of nucleotide diversity for *Angraecum* plastomes. (**A**) The nucleotide diversity of the whole plastome; five mutation hotspot regions were annotated. (**B**) The nucleotide diversity of 68 CDSs. The window size was set to 100 bp and the sliding windows size was 25 bp. *x*-axis, position of the midpoint of a window; *y*-axis: nucleotide diversity of each window or genes.

To further explore the mutation hotspots of *Angraecum* plastomes to develop specific DNA barcodes, Pi values was calculated using DnaSP6 (Figure 8). The average Pi value among the four plastomes was 0.00777, with the IR region averaging 0.00251, the LSC region averaging 0.00919, and the SSC region averaging 0.02019, respectively (Supplementary Table S6). According to the ranking of Pi values, five hypervariable regions were identified: *trnS^GCU^*-*trnG^GCC^*, *ycf1*-*trnN^GGU^*, *trnN^GUU^*-*rpl32*, *psaC*-*ndhE* and *trnS^GCU^*-*trnG^GCC^*. In terms of protein-coding genes, *rpl32*, *rps16*, *psbK*, *rps8*, and *ycf1* showed high Pi values and may be used as DNA barcodes for further phylogenetic analyses and species identification.

### 2.6. Phylogenetic Analysis

**Figure 9:**
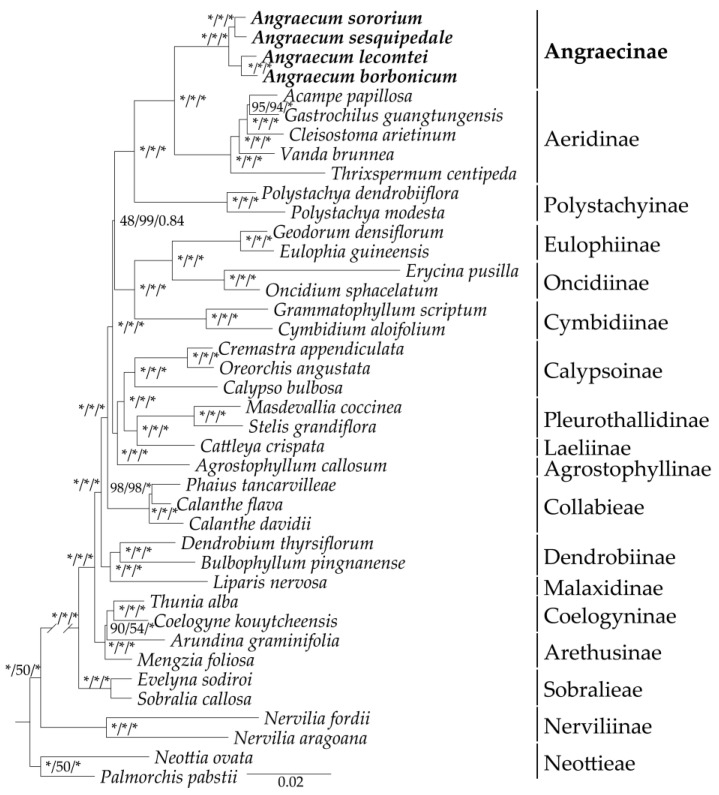
Phylogenetic tree obtained by maximum-likelihood analysis based on the complete plastome. The numbers near the nodes are bootstrap percentages and Bayesian posterior probabilities (BP_ML_, BP_MP_, PP). * node is the 100 bootstrap percentage or 1.00 posterior probability.

In the present study, we obtained a robust phylogenetic framework of the Epidendroideae using three methods (ML, MP and BI), including 40 species from 34 genera (Figure 9). The species of *Angraecum* formed a well-supported monophyletic group (BS = 100, PP = 1.00), which was revealed as a sister to Aeridinae. The intrageneric relationships of *Angraecum* showed that *Angraecum* could be divided into two diverging clades with strong support (BS = 100, PP = 1.00). The taxa *A. lecomtei*, together with *A. borbonicum*, formed the first distinct clade. *A. sororium* was grouped together with *A. sesquipedale*, supported as the second clade.

## 3. Discussion

### 3.1. The Plastome Characteristics and Structural Evolution 

In the present study, we firstly reported four *Angraecum* plastomes and provided genetic resources for understanding the evolution of plastomes in this group. All *Angraecum* plastomes had the typical quadripartite structure (Figure 1), consisting of one LSC region, one SSC region, and two IR regions, similar to most common angiosperms. Limited variation in overall plastome size was detected among *Angraecum* species: *A. borbonicum* possessed the smallest plastome at 150,743 bp, and *A. sesquipedale* had the largest at 151,818 bp. The plastome size falls within the previously reported range of Orchidaceae plastomes, which ranged from 19,047 bp (*Epipogium roseum*) [29] to 212,688 bp (*Cypripedium tibeticum*) [24]. No significant variation in GC content was found among *Angraecum* plastomes (36.7–36.9%) in this study. In addition, no unusual structural features were detected among *Angraecum* plastomes according to the result of Mauve (Figure 2).

The loss and pseudogenization of the *ndh* genes were commonly observed in Orchidaceae (Figure 1, Table 1). The phenomenon has been observed in several orchid lineages, including *Vanilla* [30], *Dendrobium* [25], *Bulbophyllum* [31], Goodyerinae [23], Neottieae [32], *Polystachya* [26], and Aeridinae [20,21]. Our study showed that all *Angraecum* plastomes were *ndh*-deleted: *ndhA*/*F*/*G*/*H*/*I* genes were completely lost and the other *ndh* genes were pseudogenes. The previous study suggests that the loss of *ndh* genes may be associated with the epiphytic lifestyle of plants [33]. *Angraecum* species are usually epiphytic or lithophytic [6], supporting the pseudogenization or loss of *ndh* genes in epiphytic habitats.

Previous studies showed that the IR/SC boundary shift is one of the main factors contributing to differences in plastome length and gene content [34,35]. However, the gene arrangement of the IR/SC boundary in *Angraecum* plastomes was extremely conserved (Figure 3), indicating that the variations in plastome length and gene content in *Angraecum* were not caused by the IR/SC boundary shift.

Simple sequence repeats (SSRs) are commonly found in plastomes, serving as a crucial molecular marker in phylogenetics, population genetics, and evolutionary studies [36,37]. The present study investigated the dispersion of repeat sequences in four *Angraecum* plastomes, which showed a similar SSR motif distribution (Figure 4). Numerous long repeat sequences were identified, with the majority falling within the range of 30 to 40 bp, consistent with ranges previously recorded in other Orchidaceae lineages [24,25,26,27,28]. However, we detected two extremely long repeat sequences within the plastome of *A. lecomtei* with lengths of 104 bp and 80 bp. This result indicated that these long repeat sequences could potentially be DNA barcodes in future studies of this species. Our results significantly contribute to understanding the development of specific DNA barcodes in *Angraecum*.

Relative synonymous codon usage (RSCU) values were used to measure codon usage bias in coding sequences, which could provide evidence for exploring the evolutionary patterns of species [38]. Our results indicated that codon usage bias was highly conserved among four *Angraecum* plastomes (Figure 5). Previous studies indicated that similar codon selection strategies may contribute to the close phylogenetic relationships between closely related species. Our results were consistent with previous studies of codon preference in Orchidaceae [26,27].

We also conducted a selective pressure analysis to compare the protein-coding genes evolution in the four *Angraecum* plastomes (Figure 6, Supplementary Table S6). The Ka/Ks ratios were crucial for understanding the adaptive evolution among species [39]. Our results showed that no genes were identified with positive selection (Ka/Ks > 1) and most genes were found to have undergone purifying selection (Ka/Ks < 1). These phenomena might reflect that most cp-genes in these *Angraecum* species were likely to undergo deleterious nonsynonymous substitutions [40].

### 3.2. Plastid Genomic Evolutionary Hotspots

To identify mutational hotspots for phylogenetic reconstruction of taxonomically problematic groups, numerous plastomes comparative analyses within Orchidaceae had been reported [24,25,26,27,28]. In this study, a total of five hotspots regions (*trnS^GCU^*-*trnG^GCC^*, *ycf1*-*trnN^GGU^*, *trnN^GUU^*-*rpl32*, *psaC*-*ndhE* and *trnS^GCU^*-*trnG^GCC^*) and five CDSs (*rpl32*, *rps16*, *psbK*, *rps8*, and *ycf1*) were selected for candidate barcodes, respectively (Figure 8). These findings may contribute to the development of specific DNA barcoding markers and the resolution of phylogenetic relationships in *Angraecum*.

### 3.3. Phylogenetic Analysis

*Angraecum* presented a considerable challenge to its phylogenetic reconstruction and classification because of the numerous disparities between morphology and molecular analyses. Based on *matK*, *trnL*-*F* and ITS, Carlsward et al. [11] revealed that *Angraecum* was non-monophyletic and exhibited collapsed relationships with low to moderate support [11]. However, based on a broader sampling and molecular markers, the phylogenetic relationships of *Angraecum*, as revealed by Micheneau et al. [12], Andriananjamanantsoa et al. [5], and Simo-Droissart et al. [13], are still unresolved due to incongruent topology and weak support. In addition, the non-monophyletic status of most sections defined by Garay [10] was commonly found in these studies [5,12,13]. Therefore, the most recent molecular phylogenetic study suggested that using genomic data to resolve the phylogeny of *Angraecum* is needed [14]. Our phylogenomic analyses revealed that the phylogenetic resolution within *Angraecum* has been greatly improved with strong support (Figure 9). Four *Angraecum* species were clustered into a monophyletic group (BS = 100, PP = 1.00) and could be further divided into two diverging clades. This result indicated that the plastome sequences were ideal molecular markers for resolving the intrageneric relationships of *Angraecum*.

According to the new Orchidaceae classification system [41], *Angraecum* was placed in the subtribe Angraecinae, sister to the subtribe Aeridinae of the subfamily Epidendroideae. Previous studies commonly indicated that Epidendroideae was the most taxonomically problematic subfamily due to its significant diversification (comprising approximately 76% of Orchidaceae species) [42]. To explore the phylogenetic position of Angraecinae, we reconstructed the phylogeny relationships within Epidendroideae, including a total of 40 species from 34 genera. Our results showed that Angraecinae was sister to Aeridinae with strong support in all analyses (BS = 100, PP = 1.00) (Figure 9), consistent with previous studies [19]. Additionally, we identified several extremely short branches within Epidendroideae (Figure 9). Short branch lengths in phylogenetic trees could be attributed to the rapid radiation events, resulting in few opportunities for molecular changes [43]. The widespread rapid radiation events among Epidendroideae may explain why the numerous species and genera within this subfamily.

## 4. Materials and Methods

### 4.1. Taxon Sampling and Sequencing

Four *Angraecum* species were selected for the study: *A*. *borbonicum*, *A*. *lecomtei*, *A*. *sesquipedale*, and *A*. *sororium*. Fresh and healthy leaf tissues of *Angraecum* were obtained from Fujian Agriculture and Forestry University (Fuzhou, Fujian, China) and Shanghai Chenshan Botanical Garden (Shanghai, China). Four *Angraecum* species were selected. Based on the previous study [41], a total of 40 plastomes from 34 genera were selected, including six species from five genera from Lower Epidendroideae as the outgroups. Voucher information and GenBank accession numbers are provided in Supplementary Table S1.

Total DNA was extracted from fresh leaves with a Plant Mini Kit (Qiagen, Redwood City, CA, USA) based on the manufacturer’s protocol, which included prewashing with STE buffer to remove inhibitory chemicals. DNA degradation and contamination were evaluated on 1% agarose gels. Next-generation sequencing (NGS) was performed on an Illumina Hiseq 4000 sequencing platform (Illumina, San Diego, CA, USA), generating 150-bp paired-end reads. Scripts were used to filter the Illumina data in the cluster with the default parameters. Paired reads were excluded from the analysis if they contained more than 50% low-quality (Q ≤ 5) bases or if the N content exceeded 10% of the reads’ base number. More than 10 Gb clean data were obtained for each species.

### 4.2. Plastome Assembly and Annotation

To obtain complete plastomes, we used a GetOrganelle pipe-line (https://github.com/Kinggerm/GetOrganelle, accessed on 1 November 2023) [44] to filter the paired-end reads with default parameters. Then, the SPAdes 3.10 [45] were employed to assemble the filtered reads. To obtain pure contigs, we further filtered the “fastg” files by the GetOrganelle script. The filtered De Bruijn graphs were then examined and corrected by Bandage [46]. Finally, four high-quality and complete plastomes were obtained.

PGA software [47] was used to annotate the newly assembled *Angraecum* plastome, and the published sequence of *Thrixspermum centipeda* (MW057769) was used as a reference. The start and stop codons in protein-coding genes were manually visualized and corrected by aligning them with the reference plastome in Geneious R11.1.5 [48]. The annotation maps were drawn using OGDRAW [49].

### 4.3. Plastome Comparative and Codon Usage Analysis

The rearrangements of *Angraecum* plastomes were identified and plotted by Mauve [50]. The genes on the boundary regions of LSC/IRb/SSC/IRa were visualized by the IRscope online program [51]. The online software MISA (http://misaweb.ipk-gatersleben.de/, accessed on 1 November 2023) [52] was employed to detect simple sequence repeats (SSRs). Parameters for SSR motifs were 10, 5, 4, 3, 3, and 3 nucleotide repeats set for mono-, di-, tri-, tetra-, penta- and hexa-motif microsatellites (mononucleotide, dinucleotide, trinucleotide, tetranucleotide, pentanucleotide, and hexanucleotide) set as the minimum threshold, respectively. The REPuter software [53] was used to detect four types of long repeat sequences, including forward (F), palindrome (P), reverse (R), and complement (C). The minimum repeat size of oligonucleotide repeats was set at 30 bp, and the Hamming distance was set at 3. Results were visualized with the R package *ggplot2* [54]. 

A total of 68 CDSs of each *Angraecum* plastome were extracted and concatenated using PhyloSuite v1.2.2 [55]. Relative synonymous codon usage (RSCU) values for each *Angraecum* species were calculated by DAMBE [56]. Finally, a heatmap was generated using TBtools [57].

### 4.4. Selective Pressure Estimation

A total of 68 CDSs were retrieved and used to investigate substitution rates, respectively. The non-synonymous (Ka) and synonymous (Ks) rates, as well as the Ka/Ks ratio, were calculated using Ka/Ks calculator ver. 2.0 [58]. When Ka/Ks > 1 indicates positive (adaptive) selection, Ka/Ks = 1 indicates neutral evolution, while Ka/Ks < 1 signifies negative (purifying) selection.

### 4.5. Sequence Divergence, Barcoding Investigation and Phylogeny

The online program mVISTA was used to analyze the diversity of *Angraecum* plastomes using the Shuffle-LAGAN [59] alignment program and *Thrixspermum centipeda* (MW057769) was used as a reference. The nucleotide variability (Pi) of whole plastomes and 68 CDSs of *Angraecum* were calculated by DnaSP 6 [60] with the default parameters.

A total of 40 plastomes were aligned by MAFFT [61] and we employed TrimAL v1.4 [62] to trim the poorly aligned positions with a default parameter. Then, the matrix was used to reconstruct the phylogenetic tree. The phylogenetic trees were inferred by maximum likelihood (ML), maximum parsimony (MP), and Bayesian inference (BI) on the website CIPRES Science Gateway web server (RAxML-HPC2 on XSEDE 8.2.12, PAUP on XSEDE 4.a 168 and MrBayes on XSEDE 3.2.7a) [63]. For ML analysis, the GTRGAMMA model was specified for all datasets [64] and calculated bootstrap values from 1000 bootstrap replicates using heuristic searches [65]. For BI analysis, we used MrBayes v. 3.2.7a under the GTR + I + Γ substitution model. The Markov chain Monte Carlo (MCMC) algorithm was run for 10,000,000 generations, with one tree sampled every 100 generations. The first 25% of trees were discarded as burn-in to construct majority-rule consensus trees and estimate posterior probabilities (PP).

## 5. Conclusions

In the present study, we firstly reported four *Angraecum* plastomes (*A. borbonicum*, *A. lecomtei*, *A. sesquipedale*, and *A. sororium*). The characteristics and comparative analysis results indicate that the genomic structure and gene content of *Angraecum* plastomes are highly conserved. All *ndh* genes were found to be lost or pseudogenized. According to the ranking of Pi values, a total of five hotspots regions (*trnS^GCU^*-*trnG^GCC^*, *ycf1*-*trnN^GGU^*, *trnN^GUU^*-*rpl32*, *psaC*-*ndhE* and *trnS^GCU^*-*trnG^GCC^*) and five protein-coding genes (*rpl32*, *rps16*, *psbK*, *rps8*, and *ycf1*) were identified for DNA barcodes. Based on whole plastome sequences, we explored the intrageneric and intergeneric relationships of *Angraecum* and found that plastome data offer valuable insights into the phylogenetic relationships of *Angraecum*. These findings shed new light on plastome evolution and the phylogenetic relationships of *Angraecum* and its related lineages.

## Figures and Tables

**Table 1:** Characteristics of the complete plastomes of *Angraecum*.

Taxa	Size (bp)	GC Content (%)	LSC Size in bp (%)	IR Size in bp (%)	SSC Size in bp (%)	Total Number of Gene	CDS	tRNA Gene	rRNA Gene	Number of *ndh* Fragment
*A. borbonicum*	150,743	36.7	88,206 (58.5)	25,387 (33.7)	11,763 (7.8)	120	74	38	8	7
*A. lecomtei*	151,277	36.8	87,889 (58.1)	25,733 (34.0)	11,922 (7.9)	120	74	38	8	7
*A. sesquipedale*	151,818	36.8	88,904 (58.6)	25,982 (34.2)	11,760 (7.7)	120	74	38	8	7
*A. sororium*	151,341	36.9	87,992 (58.1)	25,875 (34.2)	11,599 (7.7)	120	74	38	8	7

## Data Availability

All the data are provided within this manuscript and supplementary materials.

## Supplementary Materials

The supporting information can be downloaded at: https://www.mdpi.com/article/10.3390/ijms25010184/s1.
